# Direction-of-Arrival Estimation for Unmanned Aerial Vehicles and Aircraft Transponders Using a Multi-Mode Multi-Port Antenna

**DOI:** 10.3390/s24113452

**Published:** 2024-05-27

**Authors:** Sami Alkubti Almasri, Nils L. Johannsen, Peter A. Hoeher

**Affiliations:** Chair of Information and Coding Theory, Kiel University, 24143 Kiel, Germany; nj@tf.uni-kiel.de (N.L.J.); ph@tf.uni-kiel.de (P.A.H.)

**Keywords:** 6G, ACAS X, airspace safety, direction-of-arrival estimation, experimental verification, multi-mode multi-port antennas, networked sensing systems, sense and avoid, unmanned aerial vehicles

## Abstract

Increasing airspace safety is an important challenge, both for unmanned aerial vehicles (UAVs) as well as manned aircraft. Future developments of collision avoidance systems are supposed to utilize information from multiple sensing systems. A compact sensing system could employ a multi-mode multi-port antenna (M${\;\;}^{3}$PA). Their ability to radiate multiple orthogonal patterns simultaneously makes them suitable for communication applications as well as bearing and ranging applications. Furthermore, they can be designed to flexibly originate near-omnidirectional and/or directional radiation patterns. This option of flexibility with respect to the radiation characteristic is desired for antennas integrated in collision avoidance systems. Based on the aforementioned properties, M${\;\;}^{3}$PAs represent a compelling option for aircraft transponders. In this paper, direction-of-arrival (DoA) estimation using an M${\;\;}^{3}$PA designed for aerial applications is put to the test. First, a DoA estimation scheme suitable to be employed with M${\;\;}^{3}$PAs is introduced. Next, the validity of the proposed method is confirmed through numerical simulations. Lastly, practical experiments are conducted in an antenna measurement chamber to verify the numerical results.

## 1. Introduction

Since safety is a big concern in air traffic, particularly regarding the safe integration of unmanned aerial vehicles (UAVs) into airspace [1,2,3,4], the integration of supporting systems and expansion of standards and regulations are topics in continuous development. In the late 2000s, efforts were directed towards reducing the risk of midair collision. These efforts led to the definition of the so-called airborne collision avoidance system (ACAS II). The latter is an on-board supporting system, operating independently of air traffic control (ATC). It monitors the traffic in the surrounding airspace to determine a potential collision risk and alarm the flight crew [5]. As a successor to ACAS II, a family of standards called ACAS X has been introduced and is currently under development. Unlike its predecessor, ACAS X has the ability to process sensing information from different sources [6]. Sensors include cooperative and non-cooperative sensors dedicated to the detect and avoid task, also referred to as sensing and avoidance [7]. The main tasks of the sensors are to provide the slant range, bearing, and altitude of intruders, as these parameters are needed for state estimation. The information gain resulting from combining inputs from multiple sensing systems assures less uncertainty and increases awareness of the surrounding airborne traffic. This ultimately leads to a higher degree of safety in air traffic. ACAS X includes multiple underlying variants for certain classes of aircraft. Examples include the baseline system ACAS Xa, which is the successor to ACAS II and is designed for manned aircraft, and ACAS Xu, which will allow multiple sensor inputs and is optimized for unmanned airborne systems [6,8]. In addition, numerous 6G initiatives are ongoing in the context of an integrated space–air–ground network [9,10,11,12]. These investigations include UAVs [13,14].

Bearing information plays a crucial rule in the surveillance of the surrounding aerial space in the current ACAS II and upcoming ACAS X [15]. Direction finding (DF) systems rely traditionally on arrays of monopole antenna elements. Based on the reception delays between the receiving antenna elements, and given the knowledge of the geometry in which the array is arranged, the direction-of-arrival (DoA) of a response from an interrogated transponder can be estimated [16].

A different approach for the purpose of DF utilizes multi-port multi-mode antennas (M${\;\;}^{3}$PAs) [17]. Based on the theory of characteristic modes [18,19,20], M${\;\;}^{3}$PAs are capable of realizing multiple orthogonal radiation patterns on a single conducting surface [21,22,23], hence reducing weight and volume. The utilization of M${\;\;}^{3}$PAs was investigated in [24,25,26,27,28,29] for communication and ultra-high-speed communication, in [27] for IoT applications with the possibility of high connectivity, and in [30,31,32] for DF use cases. Furthermore, approaches for joint communication and sensing specifically designed towards integrating M${\;\;}^{3}$PAs aboard UAVs are presented in [33,34,35]. ACAS Xu standards [36] recommend using a directional antenna mounted on the top of the aircraft for directional interrogations of Mode C transponders. An omnidirectional antenna mounted on the bottom of the aircraft is sufficient since the top antenna is preferred for transmitting interrogations. However, a directional antenna on the bottom for Mode S and a traffic alert and collision avoidance system’s (TCAS’s) broadcast interrogations could optionally be utilized. Considering the aforementioned requirements of ACAS Xu, the ability to radiate simultaneously in both directional and omnidirectional manners, in addition to the DF abilities, M${\;\;}^{3}$PAs represent an attractive option for integration onboard aircraft, as a part of collision avoidance systems.

Hence, a significant amount of interest has recently been directed towards utilizing M${\;\;}^{3}$PAs for the purpose of solving the DF problem in aerial applications. In [37,38,39], platform-based DF system designs based on the theory of characteristic modes were presented. First, in [37], the chassis of the aircraft was used as the main radiator of an antenna array for DF purposes. The performance of the system suggested in [37] was enhanced in [38] by introducing a dynamic selection of the excited modes. In [39], a further improvement was introduced to the platform-based DF system by reducing the area on which the DF array was distributed. The last three publications had the 3–300 MHz frequency band in common. In contrast, our work does not use the chassis as the main radiator, and we aim to operate in the ACAS and Mode S frequency range between 1030 MHz and 1090 MHz.

In related work, ref. [40] presented an innovative design of an M${\;\;}^{3}$PA antenna specific for utilization in aerial application within the aforementioned ACAS and Mode S frequency range. The authors provided design guidelines from an antenna development point of view. However, the evaluation of the designed M${\;\;}^{3}$PA antenna is based only on the deterministic properties of the antenna. In other words, the suitability of the suggested design is evaluated independently of the statistical parameters that are associated with DF algorithms and their theoretical bounds such as the Cramér–Rao bound (CRB). Contrarily, the objective of this paper is to test and verify the DF capabilities of the M${\;\;}^{3}$PA designed in [40] based on fundamental DF methods. The contributions of this paper can be outlined as follows: (i) A MUSIC-based DoA estimation scheme utilizing the investigated M${\;\;}^{3}$PA is introduced. It is based on measured patterns of a prototype of the mentioned M${\;\;}^{3}$PA. (ii) The proposed approach is analyzed and evaluated by means of numerical simulations. (iii) The theoretical results are experimentally validated in an antenna measurement chamber using the M${\;\;}^{3}$PA prototype and a software-defined radio (SDR).

Accordingly, this paper is organized as follows: Section 2 provides an overview of the M${\;\;}^{3}$PA under investigation. Section 3 describes the assumed system briefly. The applied DoA estimation algorithm is introduced in Section 4. Numerical results are presented in Section 5. Section 6 delivers the measurement setup. The experimental results are shown in Section 7. A discussion is provided in Section 8. Finally, Section 9 provides a discussion and concludes the paper.

## 2. Cuboid Multi-Mode Multi-Port Antennas

M${\;\;}^{3}$PAs [17] are designed based on the theory of characteristic modes. According to this theory, the surface current on a conducting body can be decomposed into orthogonal components, called characteristic modes. Each set of these characteristic modes corresponds to a far-field pattern. Depending on the chosen M${\;\;}^{3}$PA design, multiple antenna ports are realized on a single antenna structure. Each of these ports excites a number of characteristic modes that result in electric far-field radiation. The resulting far-field radiation patterns are accordingly orthogonal and can be radiated simultaneously [22].

**Figure 1:**
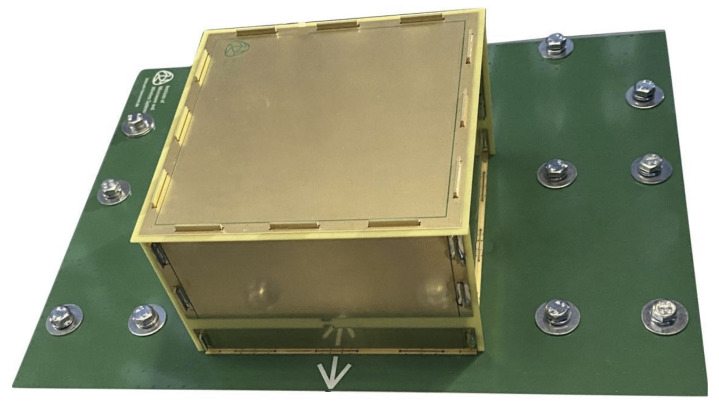
The antenna proposed in [40]. This antenna is used throughout this paper.

**Figure 2:**
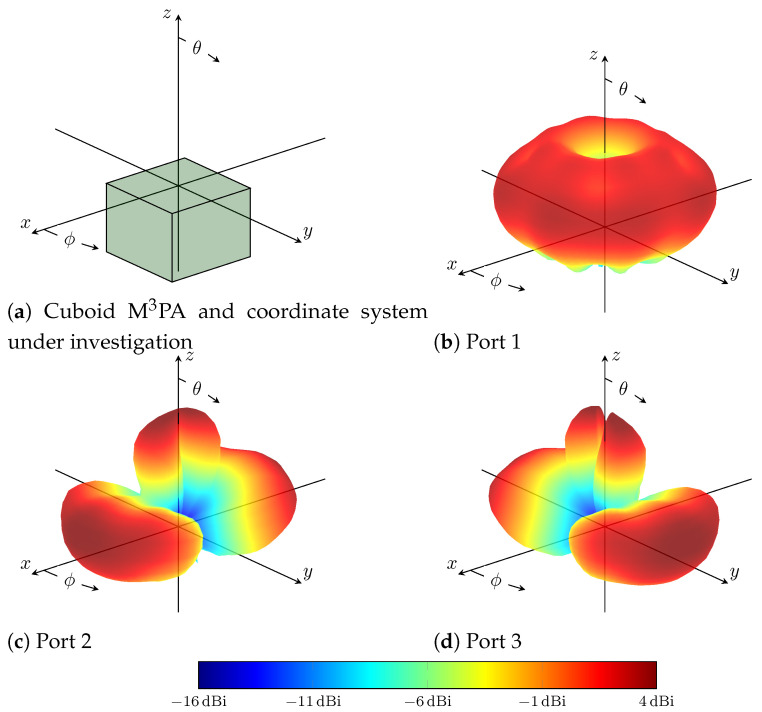
The measured θ-component’s realized gain of the three ports of the investigated M${\;\;}^{3}$PA.

An innovative M${\;\;}^{3}$PA design suitable for aerial DF applications was recently introduced in [40]. It has a cuboid-shaped structure and provides three uncorrelated ports that can be driven individually. A photo of the antenna used in subsequent simulation and experimental results is shown in Figure 1. The operating frequency is 1060 MHz. This is the central frequency between 1030 MHZ and 1090 MHZ, the operating frequencies of ACAS and Mode S interrogators [5]. The design introduced in [40] followed a systematic procedure for analyzing and developing the investigated M${\;\;}^{3}$PA in order to fulfill DF requirements in aerial applications. As suggested in [36], the employed antennas in ACAS Xu should be vertically polarized. Furthermore, omnidirectional as well as directional radiation patterns are desired for the transmission of interrogations and reception of replies. Accordingly, the implemented M${\;\;}^{3}$PA features one omnidirectional and two directional radiation patterns of the θ-component. Figure 2a illustrates the position of the cuboid M${\;\;}^{3}$PA in the considered 3D Cartesian space, along with the definition of azimuth ϕ and co-elevation θ angles. Figure 2b–d show the measured realized gain along the θ-component of the three ports of the investigated M${\;\;}^{3}$PA in the far field. The gain along the co-polarized θ-component dominates the radiation pattern of the considered M${\;\;}^{3}$PA. Therefore, the gain along the cross-polarized ϕ-component is negligible and hence not shown here.

## 3. System Description

The recommended ACAS standards towards flight transponder interrogations [36,41] suggest using a directional antenna mounted on the top of the aircraft and a monopole antenna mounted on the bottom for monitoring aircraft equipped with Mode A/C interrogators. The utilization of directional antennas on the bottom side of the aircraft is optional. Furthermore, a uniform planar array consisting of four or five monopole antenna elements, with inter-element spacing of a quarter wave, is employed for DF purposes [15]. Similar to the mentioned recommendations, our system assumes the aircraft is equipped with two cuboid M${\;\;}^{3}$PAs. One is mounted on the top of the UAV or manned aircraft, the other on the bottom. In contrast to using either monopole or directional antennas, the cuboid M${\;\;}^{3}$PA used in our system is able to provide a monopole radiation pattern and two directional radiation patterns simultaneously, while being mutually orthogonal. Hence, it can be utilized in a collision avoidance system for both transponder interrogations and DF, consequently eliminating the need for a separate planar array.

## 4. MUSIC-Based 3D DoA Estimation Adopted to Multi-Mode Multi-Port Antennas

The potential of DoA estimation using M${\;\;}^{3}$PAs was studied in [30,31,32]. Different M${\;\;}^{3}$PA prototypes were investigated in a simulation setup to estimate the DoA of impinging signals on the considered antenna. These studies focused on estimating the angle of arrival in a 2D plane using a maximum-likelihood (ML) estimator. This estimator is optimal in the sense of estimation theory, but unsuitable for practical purposes because it suffers from high computational complexity [16]. Contrary to the aforementioned publications, this paper applies the MUSIC algorithm [42] to estimate the DoA in 3D space, i.e., both azimuth (ϕ) and co-elevation (θ) angles are estimated. In the following, a quick recap of the MUSIC algorithm and its application to M${\;\;}^{3}$PAs is presented. Let $x\left(k\right)\in C^{Q\times 1}$ be the signal vector consisting of *Q* narrowband signals impinging on the M${\;\;}^{3}$PA from directions [ϕ, θ] = [($\phi_{1},\theta_{1}),...,(\phi_{Q},\theta_{Q}$)] at snapshot *k*. Given that *M* is the number of ports of the M${\;\;}^{3}$PA, then the signal $y\left(k\right)\in C^{M\times 1}$ at the output of the receiver can be written as

(1) y(k)=A(ϕ,θ)x(k)+n(k),k=1,…,K,

where *K* is the total number of snapshots. $A\left(\phi ,\theta \right)\in C^{M\times Q}$ is the antenna response matrix. It accommodates *Q* antenna response vectors $a\left(\phi_{q},\theta_{q}\right)\in C^{M\times 1}$. Each vector $a(\phi_{q},\theta_{q})$ contains the responses $a_{m}\left(\phi_{q},\theta_{q}\right)$ of *M* ports to a signal arriving from direction ($\phi_{q}$, $\theta_{q}$). Given that $g_{m}\left(\phi_{q},\theta_{q}\right)$ and $\Phi_{m}\left(\phi_{q},\theta_{q}\right)$ are the gain and phase response of port *m*, respectively, the response can be defined as

(2) $$a_{m}\left(\phi_{q},\theta_{q}\right)=\sqrt{g_{m}\left(\phi_{q},\theta_{q}\right)}e^{j\Phi_{m}\left(\phi_{q},\theta_{q}\right)}.$$

**Figure 3:**
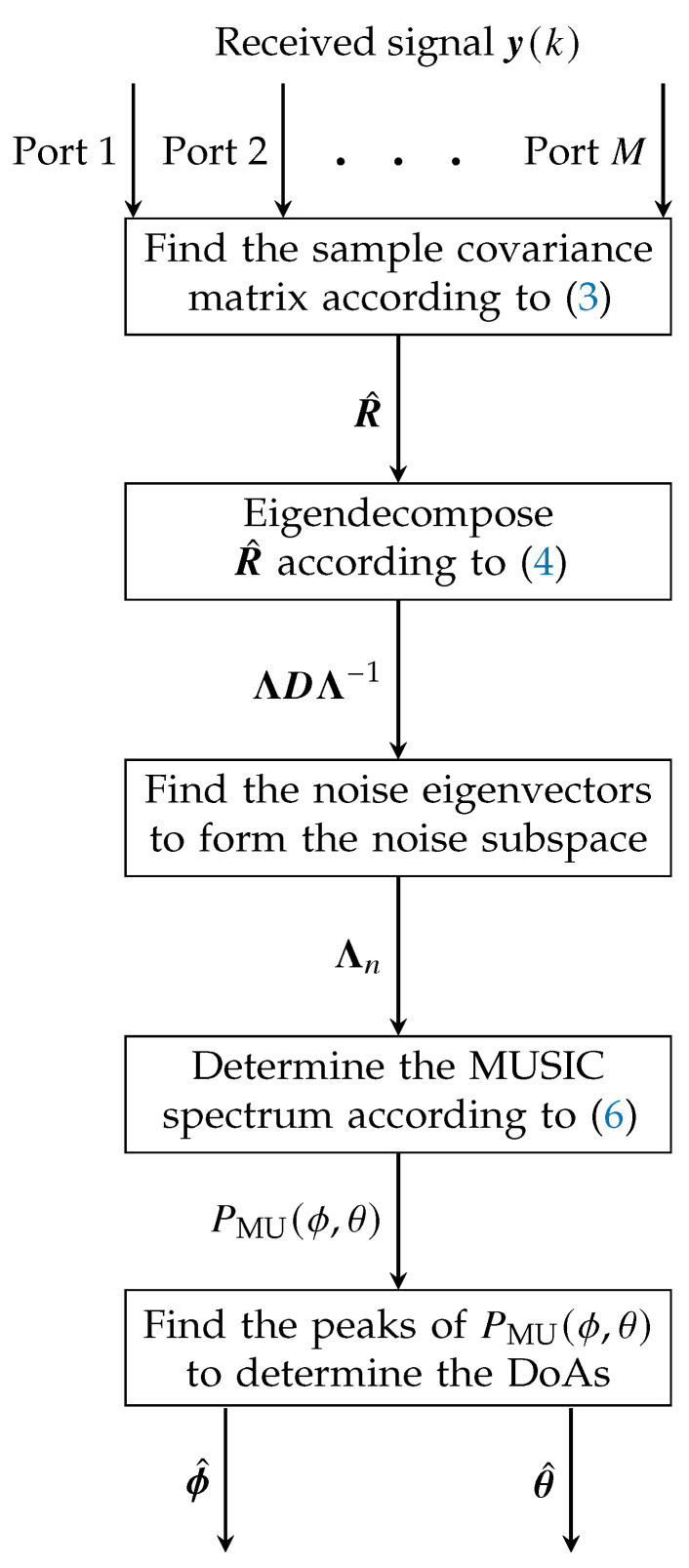
MUSIC-based 3D DoA estimation adopted to multi-mode multi-port antennas.

$n\left(k\right)\in \;C^{M\times 1}$ models a zero-mean Gaussian distributed noise process. The sample covariance matrix of y(k) is frequently defined as

(3) $$\hat{R}=\frac{1}{K}\sum_{k=1}^{K}y\left(k\right){y(k)}^{H},$$

where ${\left(\;\cdot \;\right)}^{H}$ denotes the Hermitian transpose. Applying eigendecomposition on the matrix $\hat{R}$ yields

(4) $$\hat{R}=\Lambda D\Lambda^{-1},$$

where Λ is a matrix with the eigenvectors of $\hat{R}$ in its columns and D is a diagonal matrix with the corresponding eigenvalues on its diagonal. Next, the matrix Λ can be split into two matrices $\Lambda_{s}$ and $\Lambda_{n}$. $\Lambda_{s}$ contains the *Q* signal eigenvectors that correspond to the *Q* largest eigenvalues; hence, it spans the signal subspace. $\Lambda_{n}$ contains the M−Q noise eigenvectors that correspond to the remaining M−Q smallest eigenvalues; hence, it spans the noise subspace. Since these two subspaces are orthogonal, the following equation

(5) $$a^{H}\left(\phi ,\theta \right)\Lambda_{n}=0$$

holds for all directions [ϕ, θ] = [($\phi_{1},\theta_{1}),...,(\phi_{q},\theta_{q}$)]. Consequently, the so-called MUSIC spectrum

(6) $$P_{\mathrm{MU}}\left(\phi ,\theta \right)=\frac{1}{{|a\left(\phi ,\theta \right)\Lambda_{n}|}^{2}}$$

exhibits peaks at the estimated directions of arrival [$\hat{\phi }$, $\hat{\theta }$]. Figure 3 and Algorithm 1 provide a flowchart and a pseudocode of the described algorithm, respectively.
**Algorithm 1** MUSIC-based 3D DoA estimation adopted to multi-mode multi-port antennas1:**Input:** $\;y\left(k\right)\in C^{M\times 1}$2:**Output:** $\;[\hat{\phi },\hat{\theta }]$3:$\hat{R}=\frac{1}{K}\sum_{k=1}^{K}y\left(k\right){y(k)}^{H}$4:$\hat{R}=\Lambda D\Lambda^{-1}$5:Determine the noise subspace $\Lambda_{n}$6:$P_{\mathrm{MU}}\left(\phi ,\theta \right)=\frac{1}{{|a\left(\phi ,\theta \right)\Lambda_{n}|}^{2}}$7:Find the peaks of $P_{\mathrm{MU}}$

## 5. Numerical Results

To validate the applicability of the cuboid M${\;\;}^{3}$PA for DF, Monte Carlo simulations using the algorithm presented in Section 4 were conducted before measurements were made. To that end, test signals x(k) that impinge on the cuboid M${\;\;}^{3}$PA from directions [$\phi_{s}$, $\theta_{s}$] were simulated. These directions [$\phi_{s}$, $\theta_{s}$] are the points in the far field, at which the patterns are measured in an anechoic chamber, as presented in [40]. Consequently, the term A(ϕ,θ) representing the antenna response in (1) becomes $A(\phi_{s}$,$\theta_{s})$. They cover the whole 3D space with a 5${\;\;}^{\circ }$ step size in both azimuth and co-elevation, i.e., $\phi_{s}=[0^{\circ },5^{\circ },10^{\circ },\ldots ,355^{\circ }$] and $\theta_{s}\;=[0^{\circ },5^{\circ },10^{\circ },\ldots ,180^{\circ }$]. This results in $n_{\phi_{s}}=72$ azimuth angles and $n_{\theta_{s}}=37$ co-elevation angles. Given that both poles, i.e., $\theta =0^{\circ }$ and $\theta =180^{\circ }$, represent the same point for any azimuth angle ϕ in the 3D space, the considered angles yield a total number of 2522 directions [$\phi_{s}$,$\theta_{s}$] covering the whole 3D space. For each simulation run, signals impinging from all of these directions were simulated. However, in order for the estimation error not to be limited by the $5^{\circ }$ measurement step size, a(ϕ,θ) in (6) and hence $P_{\mathrm{MU}}\left(\phi ,\theta \right)$ need to be calculated at any arbitrary direction (ϕ,θ). Therefore, the wavefield modeling (WM) technique [43,44,45] was applied to the measured antenna response $A(\phi_{s}$,$\theta_{s})$. The application of WM on M${\;\;}^{3}$PAs was thoroughly investigated in [31]. For a range of SNR values, the root mean square error (RMSE) for each estimated $\hat{\phi }$ and $\hat{\theta }$ is calculated according to

(7) $$\mathrm{RMSE}\left(\phi {\;\;}_{s}\right)=\sqrt{\frac{1}{N_{\mathrm{MCr}}}\sum_{n_{\mathrm{MCr}}=1}^{N_{\mathrm{MCr}}}{\left(\hat{\phi }{\;\;}_{n_{\mathrm{MCr}}}-\phi_{s}\right)}^{2}}\;\mathrm{and}$$

**Figure 4:**
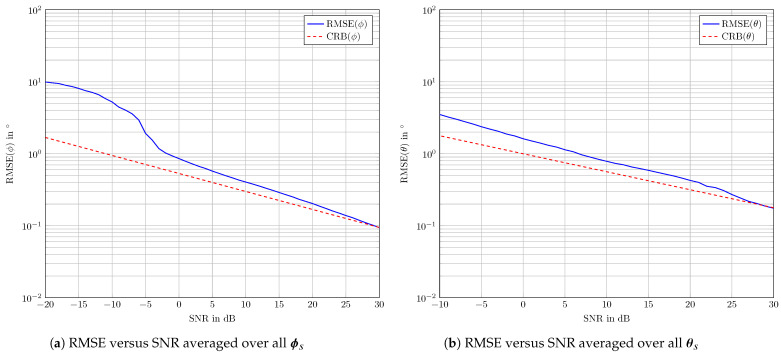
RMSE and CRB versus SNR of the estimated directions $\left[\phi_{s},\theta_{s}\right]$.

(8) RMSE(θ s)=1NMCr∑nMCr=1NMCr(θ^ nMCr−θs)2,and

respectively. $N_{\mathrm{MCr}}$ represents the number of simulation runs. As a benchmark for the quality of direction estimation, the Cramér–Rao bound (CRB) is used, as it is known to be the lower bound of any unbiased estimator [46,47]. Figure 4 shows the results of the numerical simulations for $N_{\mathrm{MCr}}$ = 1000 runs. In Figure 4a, for each SNR value, the RMSE values are averaged over all values of $\phi_{s}$. Similarly, Figure 4a depicts the averaged RMSE values over all values of $\theta_{s}$ for each SNR. It can be observed in Figure 4 that the estimation error performs close to the CRB over a large range of SNR values and converges asymptotically to the CRB at high SNRs. This demonstrates the validity of the cuboid M${\;\;}^{3}$PA under investigation in combination with the MUSIC algorithm for the purpose of direction finding.

## 6. Measurement Setup

**Figure 5:**
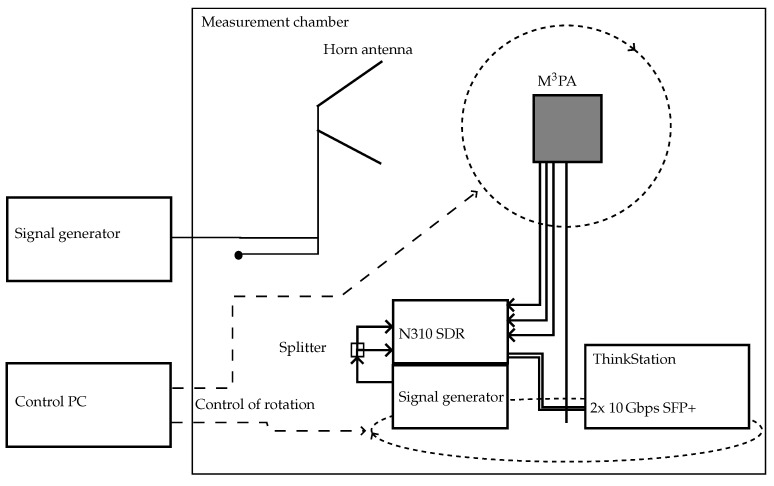
Measurement setup in antenna measurement chamber.

**Figure 6:**
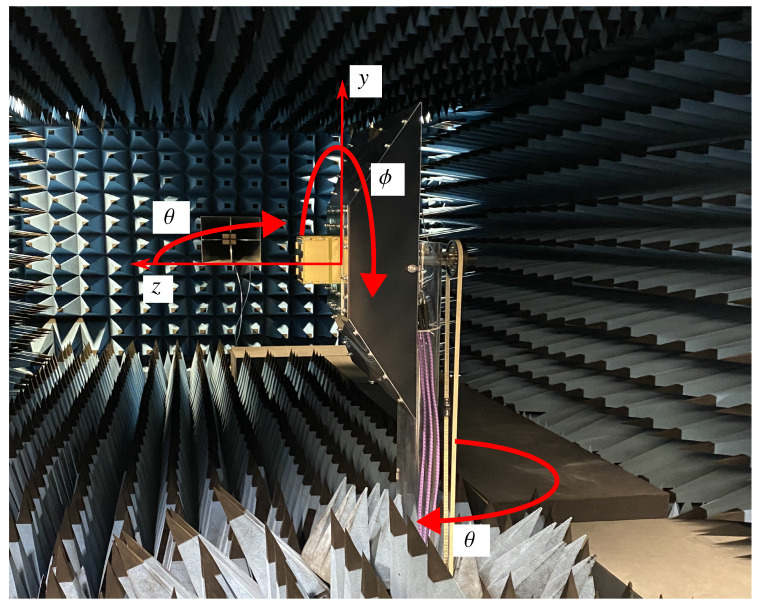
M${\;\;}^{3}$PA antenna and coordinate system in antenna measurement chamber.

In Figure 5, a block diagram providing an overview of the measurement setup is shown. The measurement is controlled from a PC outside the measurement chamber at the Institute of Microwave and Wireless Systems (IMW) at Leibniz University Hannover (LUH). It steers the rotation of the M${\;\;}^{3}$PA proposed in [40], which is mounted on a rotating arm. The corresponding antenna coordinate system and its location on the rotating arm can be seen in Figure 6. A signal generator is connected to the antenna port of the quad-ridged horn antenna used for the transmission of a vertically polarized wave. This antenna can be seen in the background of Figure 6. The transmitted signal is received by the M${\;\;}^{3}$PA. The M${\;\;}^{3}$PA is designed for a center frequency $f_{c}=1060$ MHz. This allows its usage for both the interrogation as well as the reply of the transponders at 1030 MHz and 1090 MHz. Note that the performance is adaptable to other frequencies by changing the size of the antenna. The three antenna ports of the M${\;\;}^{3}$PA are connected to three ports of the SDR. The SDR is used to sample the received signals. The SDR under consideration is an Ettus USRP N310 [48]. It provides four ports and is capable of processing signals in the frequency domain of up to 6 GHz, offering a bandwidth of up to 100 MHz. A signal generator delivers an external local oscillator (LO) signal to the SDR, which is required for phase-coherent applications, as performed in [49]. This is due to the fact that inside the SDR two independent boards are used which lack mutual phase coherency. This can be mitigated by directly feeding the same clock signal to both boards. Phase coherency is mandatory when estimating the incident angle, since the algorithms rely on the phase difference of the signal due to the radiation pattern [16]. Inside the SDR, the LO frequency of 2120 MHz is divided by two, which corresponds to an intermediate frequency (IF) of 1060 MHz. The transmission lines connecting the ports of the antenna to the SDR require an additional step of calibration. Prior to the measurements, a calibration of the signal paths needs to be conducted. An LO is used to deliver a synchronous signal via a splitter to two transmission lines connected to the ports of the SDR. The resulting differences in phases and amplitudes can than be compensated in software. The phases and attenuations of the feeding network of the antenna are known from simulations and can hence also be compensated by the SDR. A more detailed description of the calibration process can be found in [49]. For controlling the SDR and the record of the sampled signals, a Lenovo ThinkStation running an Ubuntu 20.04 with GNU Radio Companion and the Ettus UHD library is applied. Two 10 Gbps SFP+ links are used to transfer data from the SDR towards the Lenovo ThinkStation. The data streams of the sampled signals at each port are recorded in parallel by the ThinkStation via GNU Radio Companion.

## 7. Experimental Results

In order to prove the validity of the DoA estimation concept using the M${\;\;}^{3}$PA under investigation, two experiments are conducted. In the first experiment, the receiving M${\;\;}^{3}$PA is assumed to be positioned in the origin of the Cartesian coordinate system, see Figure 2a. The test signals generated by the LO are transmitted using the quad-ridged horn antenna from multiple known directions [ϕ, θ] in the far field and received using the considered cuboid M${\;\;}^{3}$PA. As explained in Section 6, the received signals are fed into the SDR and the DoAs are estimated using the MUSIC algorithm presented in Section 4. In order to demonstrate the impact of transmit power on the accuracy of the estimation, five different transmit power levels $P_{T}$ = [0 dBm, −10 dBm, −20 dBm, −30 dBm, −40 dBm, −50 dBm] are used for each of the tested directions [ϕ, θ]. The performance of the estimation is evaluated by means of the errors $\varepsilon_{\phi }$ and $\varepsilon_{\theta }$ for the azimuth and co-elevation angles, respectively. These errors are calculated as the absolute difference between the known angle and the estimated angle, i.e., $\varepsilon_{\phi }$ = |ϕ − $\hat{\phi }|$ and $\varepsilon_{\theta }$ = |θ − $\hat{\theta }|$. Furthermore, as a performance metric, the angle ψ between the position vector pointing to the known direction [ϕ,θ] and the position vector pointing to the estimated direction $\left[\hat{\phi },\hat{\theta }\right]$ is considered. This angle is a known measure, utilized for determining the orthodrome, which is the shortest distance between two distinctive points on the surface of a sphere [33]. For each conducted measurement, the angle ψ is calculated according to

(9) $$\psi =\arccos(\sin(\theta )\sin(\hat{\theta })\cos(\phi -\hat{\phi })\;+\;\cos(\theta )\cos(\hat{\theta })).$$

In the remainder of this paper, the angle ψ is called the error angle. The results of the experiment are represented in Table 1 for single-shot measurements. As can be seen in the table, with the exception of the lowest transmit power $P_{T}$ = −50 dBm, the direction estimation error is less than $5^{\circ }$ for signals arriving from directions with $\theta <90^{\circ }$. The estimation error for signals arriving from directions with $\theta =90^{\circ }$ is larger than $5^{\circ }$ in some cases. However, this is considered to be a very good estimation accuracy, since DF errors can reach up to $30^{\circ }$ in ACAS II [5]. By means of standard signal processing techniques like smoothing and tracking, the estimation error can be further reduced. Only for signals arriving from directions with $\theta >90^{\circ }$ does the estimation accuracy degrade due to the antenna’s geometry.

As is evident in Figure 2b–d, only a small gain in the vertically polarized components is realizable due to the existence of the feed network and the ground plane. Hence, a performance degradation in DoA estimation is expected for signals arriving from directions underneath the horizon. However, these signals will be received by the M${\;\;}^{3}$PA mounted on the bottom of the UAV or manned aircraft, see Section 3, allowing an accurate estimation of their DoA.

**Figure 7:**
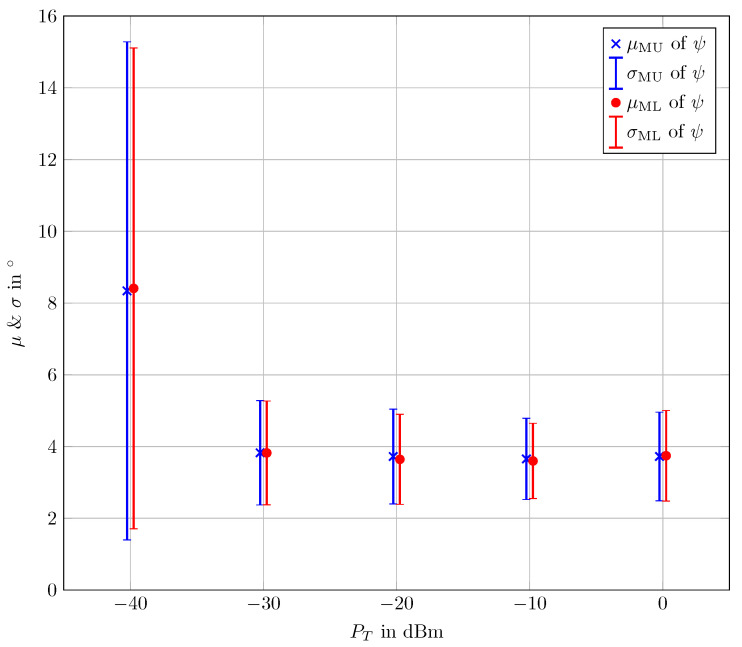
Transmit power ($P_{T}$) vs. MUSIC-based mean ($\mu_{\mathrm{MU}}$) and standard deviation ($\sigma_{\mathrm{MU}}$) of ψ and ML-based mean ($\mu_{\mathrm{ML}}$) and standard deviation ($\sigma_{\mathrm{ML}}$) of ψ.

In order to obtain a more thorough overview of the results, the mean ($\mu_{\mathrm{MU}}$) and the standard deviation ($\sigma_{\mathrm{MU}}$) of the error angle ψ are depicted in Figure 7. Furthermore, an ML estimator is applied to the experimental data to estimate the DoAs. ML estimation is optimal in the sense of estimation theory [46,50]. Thus, it serves as a fair assessment tool to evaluate the performance of the DoA estimation. The mean ($\mu_{\mathrm{ML}}$) and the standard deviation ($\sigma_{\mathrm{ML}}$) of the error angle ψ using ML estimation are also depicted in Figure 7. For both employed estimation methods, the mean value and the standard deviation in Figure 7 are calculated for signals arriving from directions with $\theta <=90^{\circ }$ for power levels $P_{T}$ = [0 dBm, −10 dBm, −20 dBm, −30 dBm, −40 dBm]. It can be clearly observed in Figure 7 that the performances of both MUSIC and ML estimators are almost identical. This proves the near-optimal estimation performance of DoA using the investigated M${\;\;}^{3}$PA. Additionally, $\mu_{\mathrm{MU}}$ and $\sigma_{\mathrm{MU}}$ remain fairly low for $P_{T}>-40$ dBm. For $P_{T}=40$ dBm, both $\mu_{\mathrm{MU}}$ and $\sigma_{\mathrm{MU}}$ increase but remain well below the tolerated error of $30^{\circ }$ in ACAS II [5].

**Figure 8:**
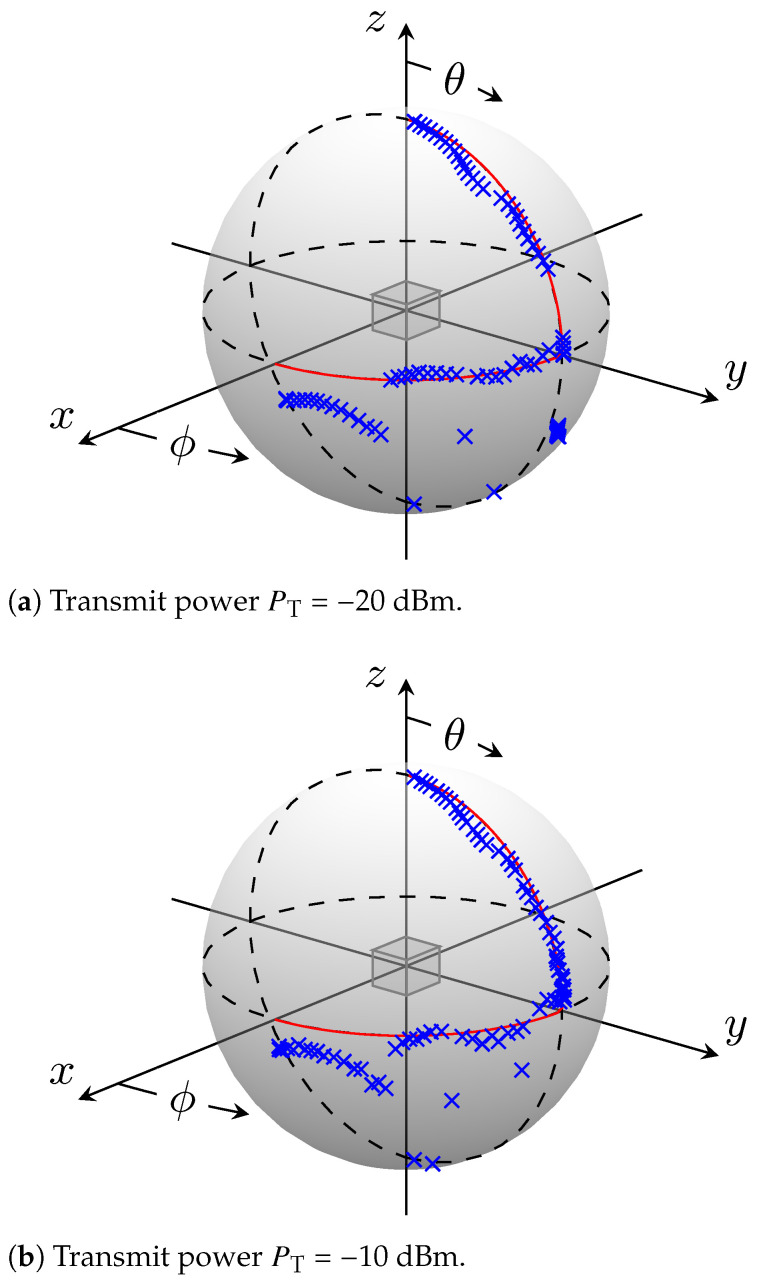
MUSIC-based DoA estimation along the traveled trajectory from ($\phi =0^{\circ }$, $\theta =90^{\circ }$) to ($\phi =90^{\circ }$, $\theta =0^{\circ }$).

In the second experiment, the M${\;\;}^{3}$PA is assumed to be positioned in the origin of the Cartesian coordinate system, similar to the case in the first experiment. The transmitting horn antenna travels while transmitting in the far field on a trajectory from direction ($\phi =0^{\circ }$, $\theta =90^{\circ }$) further on the xy-plane to direction ($\phi =90^{\circ }$, $\theta =90^{\circ }$), and it subsequently continues traveling upwards on the yz-plane to direction ($\phi =90^{\circ }$, $\theta =0^{\circ }$). This experiment is conducted using two different transmit power levels $P_{T}=[-10$ dBm, −20 dBm]. The results based on the MUSIC Algorithm are illustrated in Figure 8. The receiving M${\;\;}^{3}$PA is depicted symbolically as a cube positioned on the origin of the 3D Cartesian space. The trajectory on which the transmit antenna travels is represented as a red solid line lying on a sphere. The intention behind using a sphere in this figure is only to show the directions from which the signals are arriving to the receiver M${\;\;}^{3}$PA, and not the distance between transmitter and receiver. Remember that the far-field condition is fulfilled. Along this trajectory, the DoAs of the received signals are estimated and plotted as blue crosses. It can be seen in Figure 8 that the estimated DoAs follow the traveled trajectory with a quite good approximation for both investigated transmit power levels. With the exception of the estimated DoAs on the pole, i.e., $\theta =0^{\circ }$, the estimator shows good performance. Corresponding evaluations were also carried out for the ML estimator. The trajectories are so similar that they are omitted here.

## 8. Discussion

In view of the increasing air traffic, especially with regard to the integration of UAVs into the airspace, as well as increasing urbanization, methods for improving airspace safety are playing an ever greater role. Hence, the development of the ACAS X family started because it supports onboard processing for large as well as unmanned aerial vehicles to create a predictive situation map and to contribute to route optimization [6,8]. Towards this goal, a multitude of sensor signals will be combined. In addition, many developments have been initiated as part of 6G initiatives, particularly for small aerial vehicles like UAVs, since 6G targets 3D networks [13], i.e., integrated space–air–ground networks [9,10,11,12,14]. We use ACAS X recommendations as a baseline for our setup, but our work is neither restricted to this ATC system nor to 6G research.

Against this background, reliable high-rate data transmission and radar technology are becoming increasingly important. Joint communication and sensing is currently an important area of research [33,34,51,52,53]. However, this also implies the need for suitable antennas that can be integrated into UAVs in terms of weight, size and design, and radiation characteristics. In numerous preliminary studies, we proposed the use of M${\;\;}^{3}$PA antennas for this use case. In this research paper, for the first time, we present measurement results from an anechoic measurement chamber for the ATC scenario under investigation, supported by computer simulations. Numerical results, experimental setup, and measurement results are reported in Section 5, Section 6, and Section 7, respectively.

The numerical results proved the ability of the cuboid M${\;\;}^{3}$PA in combination with the presented MUSIC-based algorithm to theoretically perform accurate DoA estimation. The practical verification was provided by means of two experiments employing a cuboid M${\;\;}^{3}$PA prototype. In the first experiment, test signals from known directions were transmitted through a horn antenna and received via the M${\;\;}^{3}$PA under investigation. As a measure for the estimation error, the error angle ψ was taken into account, since it provides an intuitive 3D measure of the error of the estimated direction in the 3D spherical space. It can be seen from Table 1, with the exception of the lowest transmit power level, i.e., $P_{T}=-50$ dBm, that the investigated M${\;\;}^{3}$PA delivers a quite good DoA estimation performance given a wide range of SNR scenarios. The DoA estimation error is commonly less than $5^{\circ }$ for signals arriving from directions with $\theta <90^{\circ }$. For signals arriving from directions with $\theta =90^{\circ }$, the error could be larger the $5^{\circ }$, yet it remains much smaller than the tolerated error of $30^{\circ }$ in the currently in-use ACAS II [15]. Hence, the radiation pattern of the M${\;\;}^{3}$PA under investigation fulfills the ACAS Xu standard requirements [36]. In order to gain deeper insight into the results of this experiment, the mean and the standard deviation of the error angle ψ for MUSIC-based DoA estimation were compared to optimal ML DoA estimation. The comparison shown in Figure 7 takes signals arriving from directions with $\theta <=90^{\circ }$ into account. The highly similar results of this comparison indicate a near-identical performance in both methods.

Based on the design of the investigated cuboid M${\;\;}^{3}$PA shown in Figure 1 and its mounting on the body of an aircraft according to Figure 2a, the majority of the radiated power is focused towards the upper hemisphere, see Figure 2b–d. For signals arriving from directions underneath the horizon, i.e., $\theta \leq 90^{\circ }$, the performance of the DoA estimation naturally degrades, see Table 1. However, collision avoidance systems typically rely on two antennas for interrogations. One is mounted on the top and the other on the bottom of the aircraft body [36,41]. Aligned with the mentioned standards and as mentioned in Section 3, our system assumes one cuboid M${\;\;}^{3}$PA mounted on the top and another mounted on the bottom of the aircraft. Since each of these M${\;\;}^{3}$PAs provides reliable coverage of the upper hemisphere relative to its position, the whole 3D space can be covered, allowing a dependable DoA estimation performance.

The second experiment provides further confirmation of the results drawn from the first one. As a supplement of the single-shot measurements, the second experiment is conducted with the transmit antenna traveling on a trajectory while transmitting at two power levels $P_{T}=[-20$ dBm, −10 dBm]. As can be seen in Figure 8 for the MUSIC algorithm, the trajectories of the estimated DoAs, represented as blue crosses, track the real traveled trajectory, represented as a red solid line, closely. The ML estimator produced a quite similar trajectory. Hence, it was skipped here. Comparing estimation performances at both transmit power levels shows a slightly better performance at the higher $P_{T}$ = 10 dBm as expected. However, for the foreseen application, the estimation at both power levels delivers quite good results for the majority of the targeted space.

## 9. Conclusions

With the evolution of airborne collision avoidance systems from ACAS II to the ACAS X family of standards, there has been a clear shift towards integrating multiple sensing systems to enhance situational awareness and mitigate the risk of collisions. The ACAS X family targets both UAVs and manned aircraft, considering that the safe integration of UAVs into the airspace is particularly problematic. This development along with the ongoing demand for efficient and robust communication by means of compact hardware inspired the utilization of M${\;\;}^{3}$PAs for aerial applications, because this advanced antenna class is able to emulate antenna arrays at a smaller form factor and lower weight. Towards this goal, this paper introduced a direction finding MUSIC-based algorithm to estimate the DoA of received signals using an M${\;\;}^{3}$PA. The measured gain patterns of an M${\;\;}^{3}$PA designed for integration on a UAV or manned aircraft were used in a numerical simulation to prove the validity of the proposed algorithm. Furthermore, the results of the conducted numerical simulations were verified experimentally. For that purpose, measurements were conducted in an anechoic chamber, using an M${\;\;}^{3}$PA prototype and an SDR. Both numerical and experimental results show good performance of the suggested algorithm in combination with the M${\;\;}^{3}$PA under investigation. Hence, M${\;\;}^{3}$PAs represent, along with suitable signal processing techniques, a promising candidate for enhancing the safety and efficiency of airborne applications, both for UAVs and manned aircraft.

## Figures and Tables

**Table 1:** Results of single-shot DoA measurements performed in anechoic chamber.

(**a**) Transmit power $P_{T}$ = 0 dBm and $P_{T}$ = −10 dBm.						
	$P_{T}$ = 0 dBm			$P_{T}$ = −10 dBm		
(ϕ, θ)	($\hat{\phi }$, $\hat{\theta }$)	($\varepsilon_{\phi }$, $\varepsilon_{\theta }$)	ψ	($\hat{\phi }$, $\hat{\theta }$)	($\varepsilon_{\phi }$, $\varepsilon_{\theta }$)	ψ
($0^{\circ }$, $30^{\circ }$)	($2.2^{\circ }$, $27.9^{\circ }$)	($2.2^{\circ }$, $2.1^{\circ }$)	$2.3^{\circ }$	($2.3^{\circ }$, $27.8^{\circ }$)	($2.3^{\circ }$, $2.2^{\circ }$)	$2.4^{\circ }$
($0^{\circ }$, $45^{\circ }$)	($1.8^{\circ }$, $43.1^{\circ }$)	($1.8^{\circ }$, $1.9^{\circ }$)	$2.2^{\circ }$	($1.8^{\circ }$, $43.1^{\circ }$)	($1.8^{\circ }$, $1.9^{\circ }$)	$2.2^{\circ }$
($0^{\circ }$, $50^{\circ }$)	($2.0^{\circ }$, $48.3^{\circ }$)	($2.0^{\circ }$, $1.7^{\circ }$)	$2.3^{\circ }$	($2.1^{\circ }$, $48.2^{\circ }$)	($2.1^{\circ }$, $1.8^{\circ }$)	$2.3^{\circ }$
($0^{\circ }$, $60^{\circ }$)	($2.6^{\circ }$, $58.8^{\circ }$)	($2.6^{\circ }$, $1.2^{\circ }$)	$2.5^{\circ }$	($2.5^{\circ }$, $58.8^{\circ }$)	($2.5^{\circ }$, $1.2^{\circ }$)	$2.4^{\circ }$
($0^{\circ }$, $80^{\circ }$)	($3.4^{\circ }$, $78.6^{\circ }$)	($3.4^{\circ }$, $1.4^{\circ }$)	$3.6^{\circ }$	($3.4^{\circ }$, $78.7^{\circ }$)	($3.4^{\circ }$, $1.3^{\circ }$)	$3.6^{\circ }$
($0^{\circ }$, $90^{\circ }$)	($3.5^{\circ }$, $86.9^{\circ }$)	($3.5^{\circ }$, $3.1^{\circ }$)	$4.6^{\circ }$	($3.4^{\circ }$, $87.0^{\circ }$)	($3.4^{\circ }$, $3.0^{\circ }$)	$4.5^{\circ }$
($30^{\circ }$, $90^{\circ }$)	($35.8^{\circ }$, $88.9^{\circ }$)	($5.8^{\circ }$, $1.1^{\circ }$)	$5.8^{\circ }$	($35.3^{\circ }$, $88.5^{\circ }$)	($5.3^{\circ }$, $1.5^{\circ }$)	$5.5^{\circ }$
($90^{\circ }$, $90^{\circ }$)	($90.4^{\circ }$, $94.9^{\circ }$)	($0.4^{\circ }$, $4.9^{\circ }$)	$4.9^{\circ }$	($90.3^{\circ }$, $94.7^{\circ }$)	($0.3^{\circ }$, $4.7^{\circ }$)	$4.7^{\circ }$
($135^{\circ }$, $90^{\circ }$)	($131.0^{\circ }$, $90.9^{\circ }$)	($4.0^{\circ }$, $0.9^{\circ }$)	$4.1^{\circ }$	($131.0^{\circ }$, $90.9^{\circ }$)	($4.0^{\circ }$, $0.9^{\circ }$)	$4.1^{\circ }$
($180^{\circ }$, $90^{\circ }$)	($181.9^{\circ }$, $86.0^{\circ }$)	($1.9^{\circ }$, $4.0^{\circ }$)	$4.4^{\circ }$	($181.9^{\circ }$, $86.0^{\circ }$)	($1.9^{\circ }$, $4.0^{\circ }$)	$4.4^{\circ }$
($0^{\circ }$, $100^{\circ }$)	($2.8^{\circ }$, $93.4^{\circ }$)	($2.8^{\circ }$, $6.6^{\circ }$)	$7.0^{\circ }$	($2.7^{\circ }$, $93.5^{\circ }$)	($1.9^{\circ }$, $6.5^{\circ }$)	$7.0^{\circ }$
($0^{\circ }$, $120^{\circ }$)	($3.0^{\circ }$, $78.8^{\circ }$)	($3.0^{\circ }$, $41.2^{\circ }$)	$41.2^{\circ }$	($2.5^{\circ }$, $78.7^{\circ }$)	($2.5^{\circ }$, $41.3^{\circ }$)	$41.3^{\circ }$
(**b**) Transmit power −20 dBm and −30 dBm.						
	$P_{T}$ = −20 dBm			$P_{T}$ = −30 dBm		
(ϕ, θ)	($\hat{\phi }$, $\hat{\theta }$)	($\varepsilon_{\phi }$, $\varepsilon_{\theta }$)	ψ	($\hat{\phi }$, $\hat{\theta }$)	($\varepsilon_{\phi }$, $\varepsilon_{\theta }$)	ψ
($0^{\circ }$, $30^{\circ }$)	($1.6^{\circ }$, $27.9^{\circ }$)	($1.6^{\circ }$, $2.1^{\circ }$)	$2.1^{\circ }$	($1.6^{\circ }$, $27.8^{\circ }$)	($1.6^{\circ }$, $2.2^{\circ }$)	$2.2^{\circ }$
($0^{\circ }$, $45^{\circ }$)	($1.8^{\circ }$, $43.1^{\circ }$)	($1.8^{\circ }$, $1.9^{\circ }$)	$2.8^{\circ }$	($1.0^{\circ }$, $43.3^{\circ }$)	($1.0^{\circ }$, $1.7^{\circ }$)	$1.8^{\circ }$
($0^{\circ }$, $50^{\circ }$)	($1.0^{\circ }$, $48.3^{\circ }$)	($1.0^{\circ }$, $1.7^{\circ }$)	$1.7^{\circ }$	($2.0^{\circ }$, $48.2^{\circ }$)	($2.0^{\circ }$, $1.8^{\circ }$)	$2.3^{\circ }$
($0^{\circ }$, $60^{\circ }$)	($3.3^{\circ }$, $78.6^{\circ }$)	($3.3^{\circ }$, $1.4^{\circ }$)	$3.5^{\circ }$	($3.1^{\circ }$, $79.0^{\circ }$)	($3.1^{\circ }$, $1.0^{\circ }$)	$3.2^{\circ }$
($0^{\circ }$, $80^{\circ }$)	($3.4^{\circ }$, $78.6^{\circ }$)	($3.4^{\circ }$, $1.4^{\circ }$)	$3.6^{\circ }$	($3.4^{\circ }$, $78.7^{\circ }$)	($3.4^{\circ }$, $1.3^{\circ }$)	$3.6^{\circ }$
($0^{\circ }$, $90^{\circ }$)	($3.8^{\circ }$, $87.3^{\circ }$)	($3.8^{\circ }$, $2.6^{\circ }$)	$4.6^{\circ }$	($2.7^{\circ }$, $87.0^{\circ }$)	($2.7^{\circ }$, $3.0^{\circ }$)	$4.0^{\circ }$
($30^{\circ }$, $90^{\circ }$)	($35.4^{\circ }$, $88.0^{\circ }$)	($5.4^{\circ }$, $2.0^{\circ }$)	$5.8^{\circ }$	($35.9^{\circ }$, $88.0^{\circ }$)	($5.3^{\circ }$, $2.0^{\circ }$)	$6.2^{\circ }$
($90^{\circ }$, $90^{\circ }$)	($90.5^{\circ }$, $95.1^{\circ }$)	($0.5^{\circ }$, $5.1^{\circ }$)	$5.2^{\circ }$	($89.7^{\circ }$, $94.2^{\circ }$)	($0.3^{\circ }$, $4.2^{\circ }$)	$4.2^{\circ }$
($135^{\circ }$, $90^{\circ }$)	($130.7^{\circ }$, $90.9^{\circ }$)	($4.3^{\circ }$, $0.9^{\circ }$)	$4.3^{\circ }$	($129.9^{\circ }$, $92.9^{\circ }$)	($5.1^{\circ }$, $2.9^{\circ }$)	$5.8^{\circ }$
($180^{\circ }$, $90^{\circ }$)	($182.0^{\circ }$, $86.0^{\circ }$)	($2.0^{\circ }$, $4.0^{\circ }$)	$4.4^{\circ }$	($181.0^{\circ }$, $85.0^{\circ }$)	($1.0^{\circ }$, $5.0^{\circ }$)	$5.0^{\circ }$
($0^{\circ }$, $100^{\circ }$)	($2.3^{\circ }$, $92.9^{\circ }$)	($2.3^{\circ }$, $7.1^{\circ }$)	$7.3^{\circ }$	($2.9^{\circ }$, $95.3^{\circ }$)	($2.9^{\circ }$, $4.7^{\circ }$)	$5.4^{\circ }$
($0^{\circ }$, $120^{\circ }$)	($2.5^{\circ }$, $79.6^{\circ }$)	($2.5^{\circ }$, $40.4^{\circ }$)	$40.4^{\circ }$	($4.3^{\circ }$, $76.9^{\circ }$)	($4.3^{\circ }$, $43.1^{\circ }$)	$43.2^{\circ }$
(**c**) Transmit power −40 dBm and −50 dBm.						
	$P_{T}$ = −40 dBm			$P_{T}$ = −50 dBm		
(ϕ, θ)	($\hat{\phi }$, $\hat{\theta }$)	($\varepsilon_{\phi }$, $\varepsilon_{\theta }$)	ψ	($\hat{\phi }$, $\hat{\theta }$)	($\varepsilon_{\phi }$, $\varepsilon_{\theta }$)	ψ
($0^{\circ }$, $30^{\circ }$)	($357.4^{\circ }$, $25.5^{\circ }$)	($2.6^{\circ }$, $4.5^{\circ }$)	$5.0^{\circ }$	($0.7^{\circ }$, $88.9^{\circ }$)	($0.7^{\circ }$, $58.9^{\circ }$)	$58.9^{\circ }$
($0^{\circ }$, $45^{\circ }$)	($5.4^{\circ }$, $42.7^{\circ }$)	($5.4^{\circ }$, $2.3^{\circ }$)	$4.4^{\circ }$	($158.7^{\circ }$, $66.4^{\circ }$)	($158.7^{\circ }$, $21.4^{\circ }$)	$108.7^{\circ }$
($0^{\circ }$, $50^{\circ }$)	($1.4^{\circ }$, $48.4^{\circ }$)	($1.4^{\circ }$, $1.6^{\circ }$)	$1.9^{\circ }$	($251.4^{\circ }$, $167.2^{\circ }$)	($108.5^{\circ }$, $117.2^{\circ }$)	$132.8^{\circ }$
($0^{\circ }$, $60^{\circ }$)	($1.5^{\circ }$, $60.3^{\circ }$)	($1.5^{\circ }$, $0.4^{\circ }$)	$1.4^{\circ }$	($13.7^{\circ }$, $150.4^{\circ }$)	($13.7^{\circ }$, $90.4^{\circ }$)	$91.1^{\circ }$
($0^{\circ }$, $80^{\circ }$)	($1.5^{\circ }$, $79.7^{\circ }$)	($1.5^{\circ }$, $0.3^{\circ }$)	$1.5^{\circ }$	($336.2^{\circ }$, $134.5^{\circ }$)	($23.7^{\circ }$, $54.5^{\circ }$)	$58.6^{\circ }$
($0^{\circ }$, $90^{\circ }$)	($7.7^{\circ }$, $96.6^{\circ }$)	($7.7^{\circ }$, $6.6^{\circ }$)	$10.1^{\circ }$	($297.9^{\circ }$, $178.2^{\circ }$)	($62.0^{\circ }$, $88.2^{\circ }$)	$89.1^{\circ }$
($30^{\circ }$, $90^{\circ }$)	($34.1^{\circ }$, $109.4^{\circ }$)	($4.1^{\circ }$, $19.4^{\circ }$)	$19.8^{\circ }$	($21.2^{\circ }$, $137.4^{\circ }$)	($8.8^{\circ }$, $47.4^{\circ }$)	$48.1^{\circ }$
($90^{\circ }$, $90^{\circ }$)	($90.6^{\circ }$, $96.9^{\circ }$)	($0.6^{\circ }$, $6.9^{\circ }$)	$6.9^{\circ }$	($110.9^{\circ }$, $160.7^{\circ }$)	($20.9^{\circ }$, $70.7^{\circ }$)	$72.02^{\circ }$
($135^{\circ }$, $90^{\circ }$)	($126.6^{\circ }$, $95.9^{\circ }$)	($8.4^{\circ }$, $5.9^{\circ }$)	$10.2^{\circ }$	($298.4^{\circ }$, $4.6^{\circ }$)	($61.6^{\circ }$, $85.4^{\circ }$)	$94.4^{\circ }$
($180^{\circ }$, $90^{\circ }$)	($188.3^{\circ }$, $110.0^{\circ }$)	($8.3^{\circ }$, $20.0^{\circ }$)	$21.7^{\circ }$	($214.3^{\circ }$, $128.8^{\circ }$)	($34.3^{\circ }$, $38.8^{\circ }$)	$49.9^{\circ }$
($0^{\circ }$, $100^{\circ }$)	($4.0^{\circ }$, $89.9^{\circ }$)	($4.0^{\circ }$, $10.1^{\circ }$)	$10.7^{\circ }$	($328.4^{\circ }$, $163.2^{\circ }$)	($31.5^{\circ }$, $63.2^{\circ }$)	$65.8^{\circ }$
($0^{\circ }$, $120^{\circ }$)	($353.5^{\circ }$, $78.0^{\circ }$)	($6.5^{\circ }$, $42.0^{\circ }$)	$42.4^{\circ }$	($69.5^{\circ }$, $138.5^{\circ }$)	($69.5^{\circ }$, $18.5^{\circ }$)	$54.8^{\circ }$

## Data Availability

Data are contained within the article.
